# Four Wellbeing Patterns and their Antecedents in Millennials at Work

**DOI:** 10.3390/ijerph16010025

**Published:** 2018-12-22

**Authors:** Tariku Ayana Abdi, José M. Peiró, Yarid Ayala, Salvatore Zappalà

**Affiliations:** 1 Department of Psychology, University of Campania, 8100 Caserta, Italy; tarikuayana.abdi@unicampania.it; 2 IVIE & IDOCAL, University of Valencia, 46010 Valencia, Spain; jose.m.peiro@uv.es; 3 Department of Economics and Management, Pontificia Universidad Javeriana, 110111 Bogotá, Colombia; 4 Department of Psychology, University of Bologna, 40126 Bologna, Italy, salvatore.zappala@unibo.it; 5 Department of Human Resource Management and Psychology, Financial University under the Government of the Russian Federation, 125993 Moscow, Russia

**Keywords:** health, job satisfaction, wellbeing, wellbeing misalignment, Millennials

## Abstract

Literature suggests that job satisfaction and health are related to each other in a synergic way. However, this might not always be the case, and they may present misaligned relationships. Considering job satisfaction and mental health as indicators of wellbeing at work, we aim to identify four patterns (i.e., satisfied-healthy, unsatisfied-unhealthy, satisfied-unhealthy, and unsatisfied-healthy) and some of their antecedents. In a sample of 783 young Spanish employees, a two-step cluster analysis procedure showed that the unsatisfied-unhealthy pattern was the most frequent (33%), followed by unsatisfied-healthy (26.6%), satisfied-unhealthy (24.8%) and, finally, the satisfied-healthy pattern (14.3%). Moreover, as hypothesized, discriminant analysis suggests that higher levels of job importance and lower levels of role ambiguity mainly differentiate the satisfied-healthy pattern, whereas overqualification and role overload differentiate, respectively, the unsatisfied-healthy and satisfied-unhealthy patterns. Contrary to our expectations, role conflict also characterizes the satisfied-unhealthy pattern. We discuss the practical and theoretical implications of these findings.

## 1. Introduction

Employee wellbeing is a multidimensional construct covering various facets and experiences, and it has no single definition [1,2,3]. However, probably the most influential narrative on wellbeing and health in the workplace is the seminal review by Danna and Griffin [4]. After a thorough synthesis of the literature, these authors propose a theoretical framework to organize and direct future theory, research, and practice focused on wellbeing and health in the workplace. In their model, wellbeing is proposed as the broader, encompassing construct that includes two main elements of the organizational research arena. First, the model suggests including both generalized job-related experiences (e.g., job satisfaction) and more facet-specific dimensions (satisfaction with co-workers). Second, the model also suggests including general health as a sub-component of wellbeing, including mental (e.g., anxiety) or physical indicators (e.g., blood pressure). Based on this model, we study wellbeing at work by focusing on job satisfaction and mental health as main indicators of employees’ wellbeing.

*Ceteris paribus*, researchers often assume that job satisfaction and mental health are associated with each other in a harmonious way, and this assumption is solidly based on previous meta-analytical evidence. For instance, a meta-analysis of 22 studies of over 4000 workers in Hong Kong [5] and another meta-analysis of 485 studies of over 250,000 individuals [6] show that employees with high job satisfaction also show high levels of mental health. Thus, there is strong evidence that these two indicators of wellbeing may have a harmonious association in which high job satisfaction is correlated with high mental health [5,6], and the opposite may be true, that is, low job satisfaction would be associated with low mental health [6]. However, in this study we consider cases where job satisfaction and mental health are associated in misaligned ways; i.e., high job satisfaction could be associated with low mental health, and vice-versa. We first provide some examples of previous research describing these paradoxical patterns, and then we propose and clarify the aim of this study.

The first misaligned wellbeing pattern is characterized by high levels of job satisfaction and low mental health. For instance, an employee may be satisfied with his/her contribution to a new program launch and, at the same time, stressed because the program unfolds more slowly than expected [7]. Another example of this type of misalignment is an employee who occupies a high-level job position who, although enjoying greater job satisfaction, might also experience low mental health in the form of high levels of job-related anxiety [8]. This type of wellbeing misalignment may also be present when high performing employees with higher-than-average salaries have high job satisfaction but also higher levels of job-demands, leading to emotional exhaustion and low mental health [2].

The second misaligned wellbeing pattern is characterized by low levels of job satisfaction and high mental health. A situation illustrating this second scenario might be the case of overqualification. Researchers have shown that overqualified employees, although reporting low levels of job satisfaction in terms of payment, growth, and promotion opportunities or incentives, also report high levels of life satisfaction, which is an indicator of mental health [9,10,11]. As such, this is a counterintuitive situation and contrasts with the concept of wellbeing spillover, which suggests that the work-domain and family-domain have similar effects on each other [12], and that low levels of job satisfaction should be related to low levels of life satisfaction or mental health.

Together, these two misaligned wellbeing patterns challenge the concept of wellbeing spillover. At the same time, they also challenge the idea that wellbeing at work should be more responsive to conditions and activities in the work-domain, and that context-free wellbeing should be more responsive to health or family-domains [13]. Paradoxically, what these misaligned wellbeing patterns suggest is that specific conditions, activities, or situations at work may simultaneously and independently impact several work-domain (e.g., job satisfaction) or context-free (e.g., mental health) aspects of wellbeing.

Therefore, the main aim of this study is to make a theoretical contribution to the understanding of misaligned wellbeing patterns. To accomplish this research aim, we propose two specific research objectives. The first objective involves the empirical identification of four wellbeing patterns. We argue that we can identify the four just mentioned wellbeing patterns by combining job satisfaction and mental health; they are: the satisfied-healthy pattern (both job satisfaction and mental health are optimized); the unsatisfied-unhealthy pattern (neither job satisfaction nor mental health are optimized); the satisfied-unhealthy pattern (job satisfaction is optimized, but not mental health); and the unsatisfied-healthy pattern (job satisfaction is not optimized, but mental health is). The second specific research objective involves identifying organizational and personal antecedents that characterize and differentiate each of the four patterns. Based on the model of health and wellbeing in the workplace, proposed by Danna and Griffin [4], we consider organizational stress (in terms of role stress and overqualification) and personal factors (in terms of job importance) as possible antecedents of the four wellbeing patterns. In Table A1, we list the constructs definitions and their relationship with employees’ wellbeing. In the following, we argue on the role they may have on the mis/aligned wellbeing patterns.

### 1.1. Role Stress

Job-related role stress has been a topic of concern across multiple disciplines [14]. Role stress can involve role conflict, role ambiguity, and role overload. Here, we briefly introduce how these components are related to job satisfaction and mental health at work.

Role conflict occurs when an employee receives contradictory or incompatible requests from different parties, or when an employee needs to produce results in different contradictory aspects. Role ambiguity occurs when employees may not have clear information about tasks required by their roles, which makes them feel uncertain about what actions to take. In today’s workplace context, role conflict and role ambiguity are salient characteristics in organizational settings. For instance, in complex organizational environments (e.g., digitalization, job redesign, multicultural works), employees are constantly required to fulfill multiple expectations and organizational roles that are ambiguous and/or contradict each other [15,16,17]. Meta-analytic evidence shows that conflicting and ambiguous roles correlate with low job satisfaction and low health [18], corroborating the role theory, which states that role conflict and role ambiguity will lead to job dissatisfaction and anxiety [19]. However, the strength of the effects of role ambiguity and role conflict on job satisfaction and mental health, although significant, may not be same. Miles [20] indicated that role ambiguity has stronger effects than role conflict on job satisfaction and mental health. Consistent with this finding, we argue that the stronger effect of role ambiguity, compared to role conflict, on the unsatisfied-unhealthy pattern is still pending confirmation. However, we do know that both role ambiguity and role conflict are significantly and negatively related to job satisfaction and mental health.

Role overload occurs when employees have too much work to do within a limited time or with limited resources, which increases the demands they must deal with. Role expansion theory states that multiple roles are beneficial for the individual because the positive effects of strong engagement in both paid work and family life outweigh the possible stressful effects on wellbeing [21]. Thus, engaging in various roles (role overload), although depleting mental health, might have positive outcomes for employee job satisfaction in terms of earning extra income, privilege, and status security [21,22], which would be related to the satisfied-unhealthy wellbeing pattern. However, further research is needed to empirically confirm whether role overload is positively related to job satisfaction, but negatively related to mental health wellbeing.

#### 1.1.1. Job Importance

Some scholars have shown that job importance, as an antecedent of employee wellbeing patterns, is associated with high job satisfaction and life satisfaction [23]. Being satisfied with life may also be related to positive mental health [10], which may predict the satisfied-healthy wellbeing pattern. More specifically, studies have shown that jobs that provide employees with job facets that are important to them can enhance their job satisfaction and decrease stress [24]. Therefore, we argue that jobs that provide employees with intrinsic, extrinsic, and social job importance facets enhance job satisfaction and mental health. Accordingly, based on role theory and empirical evidence on job importance, we hypothesize that:

**H1**: *Role conflict, role ambiguity, and job importance will mainly differentiate between the unsatisfied-unhealthy and the satisfied-healthy patterns*.

**H2**: *High role overload will characterize employees with the satisfied-unhealthy wellbeing pattern*.

#### 1.1.2. Overqualification

Nowadays, overqualification is ubiquitous across European job markets, especially in Italy and Spain, and even more so among younger employees [25,26,27]. Beyond its ubiquity, overqualification raises concerns due to its negative effects on job satisfaction. For instance, studies conducted on young Spanish and Italian employees show that overqualified employees have lower job satisfaction [9,26,28].

Some scholars explain the negative effect of overqualification on job satisfaction based on equity theory. According to equity theory, employees compare the resources they put into work (such as level of education, skills, knowledge, experience) to what they receive in return (e.g., payment, recognition, or responsibility), in order to determine their sense of fairness [9]. When they perceive that their input is greater than what they receive, they develop a sense of unfairness, and as a result, they experience dissatisfaction with their job. However, some studies have shown that, although overqualification has a negative relationship with job satisfaction, at the same time, it has a null or positive indirect relationship with mental health [10,26]. Therefore, we argue that overqualification might negatively affect employees’ job satisfaction, but not necessarily their mental health. Furthermore, a study in German firms on the effects of overeducation on productivity, comparing employees working in jobs with similar levels of requirements, observed that overqualified employees are found to be healthier and strongly work- and career-minded [29]. Therefore, we also hypothesize that:

**H3**: *Overqualification will mainly discriminate employees with the unsatisfied-healthy pattern from the rest of the patterns*.

**Figure 1:**
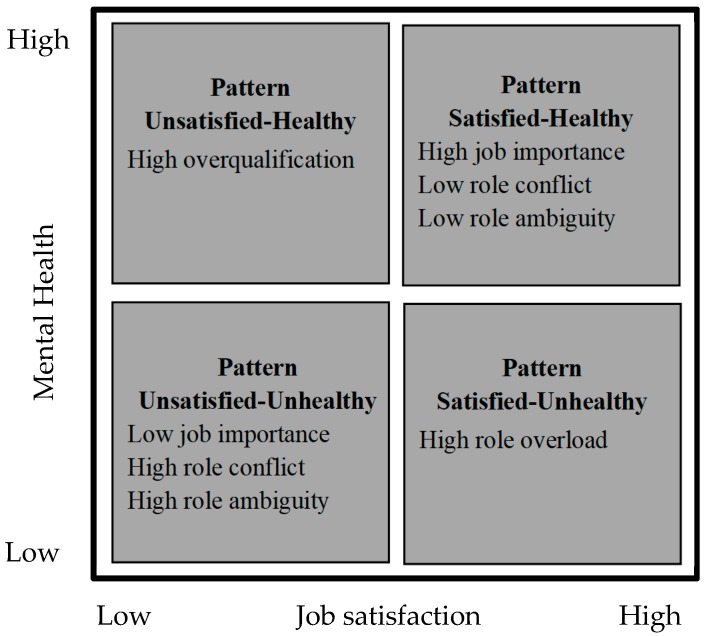
Research Model.

We test our hypotheses in a sample composed of young employees born between 1980 and 2000, typically called Millennials [30]. Knowledge about individual key outcomes such as wellbeing and health in young employees is still limited and deserves the attention of researchers and practitioners [31]. At the same time, there are approximately 1.8 billion millennials around the world. In 2018, they represent nearly 50% of the global workforce [32]. Therefore, improving outcomes for youth is fundamental to building more inclusive and sustainable societies [33], and one way to this is by promoting full and productive employment and decent work for all […] including young people […], which is part of the global agenda of the Sustainable Development Goals [34]. Figure 1 summarizes the four wellbeing patterns resulting from job satisfaction and mental health and the five antecedents we are considering.

## 2. Materials and Methods

### 2.1. Study Design and Procedure

Data were collected from a survey on the transition of young employees to the labor market, which is part of the Valencian Institute of Economic Research (IVIE in Spanish). The survey was designed to facilitate the socioeconomic and psychosocial analysis of young employees’ transition to the labor market. Participants between 16 and 30 years old who had been looking for or had found a job in the past 5 years were randomly selected for this study and then contacted by telephone. After two attempted contacts, the researchers replaced non-respondents with a randomly chosen substitute of the same age and gender. Considering the aims of this study, we focused only on respondents who were currently employed. Employees contacted by telephone were always informed of the purpose of the study and assured of the confidentiality of the data. Those who gave their consent to take part in the research were interviewed in their homes using a structured face-to-face procedure.

### 2.2. Participants

In all, 783 young Spanish respondents were selected for this study. This sample is representative of all the regions in Spain. As mentioned above, the ages of the participants ranged from 16 to 30 years old (*M*_age_ = 25.21, *SD* = 3.40), with slightly more females (52%). Most of the participants worked in the private sector (82%), and most of them had a temporary contract (58%).

### 2.3. Variables/Instruments

Job satisfaction was assessed as the composite of extrinsic, intrinsic, and social job satisfaction [35]. This measure can be applied to a wide range of jobs. Extrinsic job satisfaction was measured with seven items. A sample item is: “Indicate your level of satisfaction with your schedule”. Intrinsic job satisfaction was measured with seven items. A sample item is: ‘‘Indicate your level of satisfaction with the variety of tasks to perform’’. Finally, social job satisfaction was measured with five items. A sample item is: ‘‘Indicate your level of satisfaction with your coworkers’’. All items were scored on a 5-point Likert scale (from 1 = *not at all* to 5 = *very much*). The three subscales had good reliability, α = 0.86 (extrinsic job satisfaction), α = 0.91 (intrinsic job satisfaction), α = 0.80 (social job satisfaction), and α = 0.94 (for the composite of the three subscales).

Items measuring health belong to the scale of the General Health Questionnaire, developed by Banks [36] in young community sample. The reliability of the 12 items reported by Banks [36] was α = 0.76. In the current study, we applied four items with higher factor loadings to measure employees’ health. A sample item is: “In the last few weeks I have noticed being constantly overwhelmed and under stress”. The respondents answered on a 5-point Likert scale (from 1 = *strongly disagree* to 5 = *strongly agree*). The scale showed good reliability (α = 0.76).

Job importance was assessed as the composite of extrinsic, intrinsic, and social job importance [37]. We chose 19 items to measure job importance provided by England and Harpaz [37]. Items were preceded by the phrase “Please, indicate the importance that each of the following aspects of the work has for you”; sample items for each facet are: “Security at work”; “Useful work for society”; “Meaningful work that makes sense to do.” The respondents answered on a 5-point Likert scale (from 1 = *nothing* to 5 = *a lot*). The scale showed good reliability (α = 0.89).

Role ambiguity was measured with the scale provided by Rizzo et al. [19]. The original reliabilities, reported by Rizzo and colleagues in two different samples were good (α = 0.82). In the current study, we selected three items with higher factor loadings to measure role ambiguity. A sample item is: “I know how and what my responsibilities and competencies are at work”. The respondents answered on a 5-point Likert scale (from 1 = *strongly disagree* to 5 = *strongly agree*). We performed reverse-scoring of the three items. This scale showed good reliability (α = 0.80).

Role conflict was measured with the scale provided by Rizzo et al. [19]. The original reliabilities in two different samples were α = 0.82. In this study, we selected three items with higher factor loadings to measure role conflict. A sample item is: “I receive incompatible requests from two or more people”. The respondents answered on a 5-point Likert scale (from 1 = *strongly disagree*, to 5 = *strongly agree*). This scale also showed a good reliability (α = 0.75).

Role overload was measured with the scale of perceived work overload, proposed by Cooke and Rousseau [38]. In the current study, we selected three items with higher factor loadings. A sample item is: “I have too much work to do everything well”. The respondents answered on a 5-point Likert scale (from 1 = *strongly disagree* to 5 = *strongly agree*). This scale showed a good reliability score in this study (α = 0.82).

Overqualification was measured with the item: ‘‘If an individual had to perform your job, what level of education would you recommend him or her to have?’’ Participants responded on a 12-point scale of the International Standard Classification of Education—ISCED ((from 1 = no studies (ISCED level 1) to Doctorate (ISCED level 12)). We also considered the individual level of education and transformed both the recommended level of education and the individual level of education into years of education. To determine whether an employee was overqualified, the recommended level of education was subtracted from the level of education achieved. Negative and zero scores were considered indicators of education under-qualification and match, respectively, and positive scores were considered indicators of overqualification [25]. In our study, 21.7% of the participants were overqualified, which is similar to the rate (21.5%) of overqualification across Europe [25].

To eliminate some alternative explanations, we considered some variables that could affect our outcome variables and therefore we controlled for gender (0 = male, 1 = female), type of sector (0 = private, 1 = public), type of employment/contract (0 = temporal, 1 = permanent), and age (in years). We describe in detail the choices and procedures related to the control variables in order to ensure transparency and facilitate the reproducibility of the results [39].

In terms of gender, previous studies show that relatively to men, women tend to report higher levels of depression, but that the positive relationship between the efforts to fulfill work role demands (which interfere with employee’s ability to fulfill family demands) and depression is stronger among men [40]. In terms of type of sector and age, previous studies also show that public organizations are good in fulfilling their promises to young employees i.e., their psychological contract, and that this is translated into improved job satisfaction [41]. Considering the type of contract, previous studies also suggest that permanents as compared with temporaries engage more in relational psychological contracting, therefore, when this is violated (e.g., by producing job insecurity), this compromises more the job satisfaction for permanents than for temporaries [42]. Finally, previous research [43] also shows that temporary employees report higher wellbeing (e.g., mental health).

### 2.4. Data Analysis

To identify the four wellbeing patterns, we performed cluster analyses. One of the advantages of using cluster analysis is that unlike other methods that emphasize the relationship among variables, clustering involves sorting cases or variables according to their similarity in one or more dimensions and producing groups that maximize within-group similarity and minimize between-group similarity [44]. Therefore, to identify the four groups of wellbeing patterns, the 783 employees were clustered based on their individual levels of job satisfaction and mental health, applying a two-step cluster analysis procedure. Loglikelihood measured the distance between job satisfaction and mental health. The clustering criterion was Schwarz’s Bayesian Criterion (BIC). Finally, to balance the distribution of responses on the job satisfaction and mental health variables, we standardized these two variables to *Z*-scores (*M* = 0, *SD* = 1) before performing the cluster analyses.

To test our hypotheses, we employed discriminant analysis to test the unique differentiating role of stress (role ambiguity, role conflict, and role overload), job importance, and overqualification across the four patterns. We conducted a stepwise solution to remove variables that did not make a unique contribution to the predictive and discriminatory function at a probability of 0.05 or less. The stepwise criterion was minimization of Wilks’ lambda. Similar studies analyzing wellbeing profiles [45] applied discriminant analysis considering Wilks’ lambda stepwise minimization criteria, as we did in the current study. Discriminant analysis is a method used in a multi-group setting to find out if a set of independent variables (nominal and/or continuous) are related to group membership and how they are combined to better understand group differences [46]. In fact, various authors suggest the use of cluster analysis in combination with discriminant analysis for further validation of clusters [47].

## 3. Results

### 3.1. Descriptive and Preliminary Analyses

We present the summary of descriptive statistics and bivariate correlations for all the variables included in this research in Table 1. Considering preliminary analyses, missing data, which can occur due to nonresponse to some questions, are a common problem in organizational research [48]. Fichman and Cummings [48] argue that improper treatment of missing data (e.g., listwise deletion, mean imputation) could lead to biased statistical inference using complete case analysis statistical techniques. However, given that we have a reasonably large sample size (*n* = 783), and considering that the percentage of missing data was rather small (less than 1%), we concluded that the missing data had no effect on the results of our study [46].

### 3.2. Cluster Analysis

**Figure 2:**
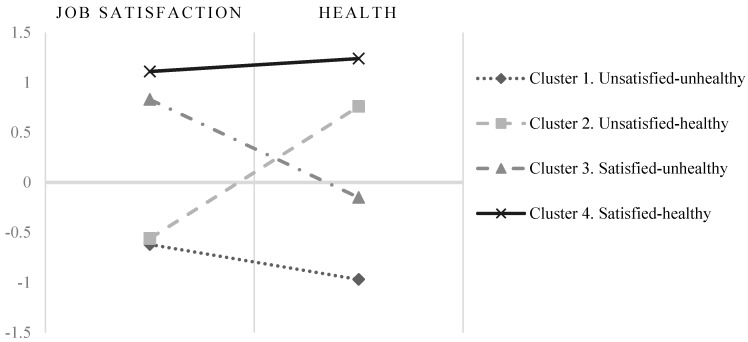
Four-cluster solution: standardized cluster means.

With five cases identified as outliers and six cases registered as missing from the system, the two-step cluster analysis efficiently and automatically formed four clusters. Following the recommendations of Aguinis, Gottfredson, and Joo [49] about the best practices for defining, identifying, and handling outliers, we defined them as cluster analysis outliers. We handled them by performing the rest of the analyses (e.g., discriminant) with and without them. We found that they were non-influential outliers because they did not significantly change the rest of our results. Figure 2 depicts the centroids (means) of each cluster, expressed in standardized scores of job satisfaction and mental health measures. The silhouette coefficient (which was approximately 0.5) suggested that a four-cluster solution had fair levels of cohesion and separation. We named the four clusters, considering the centroids of job satisfaction and mental health. Cluster 1 was called unsatisfied-unhealthy and comprised 33% of the sample (258 employees), showing the lowest means on job satisfaction (−0.62) and mental health (−0.97). Cluster 2 was called unsatisfied-healthy and comprised 26.6% of the sample (208 employees), showing low levels of job satisfaction standardized means (−0.56), but high levels of mental health (0.76). Cluster 3 was called satisfied-unhealthy and comprised 24.8% of the sample (194 employees), in this case showing high levels of job satisfaction (0.83), but low levels of mental health (−0.15). Finally, Cluster 4 was called satisfied-healthy and comprised only 14.3% of the sample (112 employees), showing the highest levels of both job satisfaction (1.11) and mental health (1.24). To test whether the clusters were significantly different from one another, we conducted an analysis of variance (ANOVA). The results suggested that there were significant differences in job satisfaction (*F*_(4, 772)_ = 276.41, *p* < 0.01) and health (*F*_(4, 772)_ = 477.93, *p* < 0.01) among the four patterns. Tukey post-hoc analyses also suggested that all the clusters were significantly different from each other. Together, these results reflect different patterns of the relations between job satisfaction and mental health.

### 3.3. Discriminant Analysis

We present the summary of the results of the discriminant analysis in Table 2. The results show that employees with the unsatisfied–unhealthy pattern (Cluster 1) have systematically higher means on role ambiguity and lower means on job importance, compared to employees with the satisfied-healthy wellbeing pattern (Cluster 4) and the rest of the patterns. Therefore, we partially confirmed hypothesis 1. Thus, role ambiguity and job importance strongly differentiated between the unsatisfied-unhealthy and satisfied-healthy patterns, but we failed to confirm role conflict. Contrary to our expectations, role conflict mainly characterized satisfied-unhealthy employees. Discriminant results also show that employees with the satisfied-unhealthy pattern (Cluster 3), systematically had significantly higher means on role overload (and role conflict), compared to employees with the satisfied-healthy pattern (Cluster 4). Therefore, we confirmed hypothesis 2, which stated that role overload characterized employees with the satisfied-unhealthy pattern. Finally, employees with the unsatisfied-healthy pattern (Cluster 2) have significantly higher means on overqualification (in comparison with the rest of the patterns) and lower means on role conflict, compared to the satisfied-unhealthy pattern (Cluster 3). Therefore, we also confirmed our hypothesis 3; thus, employees with the unsatisfied-healthy pattern perceived themselves as more overqualified for the job/position compared to their satisfied-unhealthy counterparts and the rest of the patterns.

Together, the results of the discriminant analyses suggest that job importance, role ambiguity, role conflict, role overload, and overqualification help to differentiate among the four patterns of relations between job satisfaction and mental health. When comparing these variables, job importance and role ambiguity were better at differentiating employees with the unsatisfied-unhealthy pattern from those with the satisfied-healthy pattern. Overqualification and role conflict were better at differentiating between employees with the unsatisfied-healthy and satisfied-unhealthy patterns.

## 4. Discussion

The main aim of this study was to contribute to the theoretical understanding of misaligned wellbeing patterns by considering the profiles emerging from the combination of different levels of job satisfaction and mental health. To accomplish this aim, we pursued two research objectives. The first was to identify four patterns of employee wellbeing based on a configurational variable that combines job satisfaction and mental health. Second, we examined some antecedents that can discriminate each of the four patterns. The antecedents we considered were role stress (role ambiguity, role conflict, and role overload), job importance, and overqualification.

Results showed that hypothesis 1 was partially confirmed. We confirmed that role ambiguity and job importance strongly differentiate between the unsatisfied-unhealthy and satisfied-healthy patterns and the rest of the patterns. On the one hand, our findings are aligned with previous studies on role ambiguity showing that the strength of relationships between role ambiguity, and job dissatisfaction and tension/anxiety are generally stronger than those for role conflict [20], whereas other studies have also shown that role ambiguity has adverse effects on employee job satisfaction and mental health [18,19,50]. On the other hand, with this hypothesis, we also confirmed that employees who perceive various facets of job importance, such as intrinsic (e.g., learning opportunity), extrinsic (e.g., job security), and social (e.g., societal contribution) facets, have optimal job satisfaction and mental health, compared to employees who have high role ambiguity. This result is consistent with previous studies [24].

Contrary to our expectation that role conflict would differentiate employees with the unsatisfied-unhealthy pattern from those with the satisfied-healthy pattern, our results instead showed that role conflict, along with role overload, characterized employees with the satisfied-unhealthy pattern. This result partly supports our hypothesis 2, confirming that role overload characterizes the satisfied-unhealthy pattern. Thus, role conflict and role overload had negative consequences on mental health, but less on job satisfaction. These results corroborate role expansion theory, which asserts that employees who engage in multiple roles at the same time receive incentives, status security, and position increments, which in turn have a positive effect on job satisfaction [21,22]. Furthermore, this study also confirms that focusing only on negative consequences of role stress is just one side of the issue, as asserted by McGowan et al. [50]. As the Job-Demand Control model indicates, highly demanding jobs can provide high decision latitude, control, and autonomy for employees, which, in turn, may decrease the negative effects of job demands on job satisfaction, although they can still produce negative effects on health [50].

Finally, we confirmed our hypothesis 3. Our argument was that overqualification would characterize employees with the unsatisfied-healthy wellbeing pattern. Results show that employees with higher levels of overqualification were characterized by job dissatisfaction and, at the same time, showed optimal levels of mental health. They may perceive that the salary, incentives, and other resources they receive from their work are not fair, given their qualifications. This argument substantiates equity theory. According to equity theory, employees compare the resources they put into the work (such as level of education, skills, knowledge, experience, etc.) to what they receive in return (such as payment, recognition, responsibility, etc.) in order to determine their sense of fairness [9]. If they perceive unfairness in what they receive, they may be dissatisfied with their job. Previous studies also have shown that overqualified employees have low job satisfaction [9,26] but higher satisfaction with their life and better mental health [6,10].

We also accomplished our first specific research objective, which was to identify these four wellbeing patterns involving job satisfaction and mental health. Surprisingly, the most populated cluster was the unsatisfied-unhealthy pattern, and more than half of our sample had a misaligned pattern i.e., either satisfied-unhealthy or unsatisfied-healthy. Traditionally, job satisfaction and mental health are believed to be harmoniously and positively correlated, with high (or low) job satisfaction positively correlated with high (or low) mental health. However, this might not always be the case. In this research, we focused on a new research paradigm by studying the combinations of different levels of job satisfaction and mental health. By combining different levels of job satisfaction and health, we identified four important wellbeing patterns and their antecedents. We especially focused on the anomalous or misaligned wellbeing patterns (satisfied-unhealthy or unsatisfied-healthy) as new typologies. Therefore, we believe that our research findings may motivate scholars to investigate the wellbeing patterns by using the current model as a framework. Furthermore, future studies could also combine the effects of role ambiguity, role conflict, role overload, job importance, and overqualification to move towards more generalizable empirical findings and theory development.

The results that job satisfaction and mental health together form four wellbeing patterns, indicate the need for theoretical precision; it is in fact important to integrate this complexity into the harmonious relationship between job satisfaction and mental health in order to study a broader taxonomy of relations and the conditions in which these patterns are elicited. For instance, our study questions the model of health and wellbeing in the workplace by Danna and Griffin [4], suggesting that it should be integrated with the four patterns of wellbeing and mental health described here. At the same time, our study questions whether wellbeing spillover always happens. Wellbeing spillover proposes that work and family domains of wellbeing have similar effects on one another [12]; therefore, we would expect low levels of job satisfaction to be related to low levels of mental health. Although our study suggests that this may be the case for the satisfied-healthy and unsatisfied-unhealthy patterns, and their antecedents in terms of role ambiguity and role importance, our results also suggest that this spillover may not always take place because spillover may not be present in the misaligned patterns. The results on their antecedents also suggest that work-related conditions and activities may affect work-domain (e.g., job satisfaction) and context-free (mental health) wellbeing at the same time, which challenges the idea that context-free wellbeing should be more responsive to health or family-domains [13]. We have already described why role conflict, role overload, and overqualification are separately related to the misaligned patterns, but we identified an alternative interaction explanation. For instance, in the introduction of this paper we argued that young employees who are overqualified may not have worse mental health because they may have jobs that do not stress them and that are viewed as stepping-stones to help them achieve higher goals (such as finishing college), all of which lead to the unsatisfied-healthy pattern. Aligned with this idea is that these employees also showed lower levels of job importance, thus confirming previous studies that showed that overqualified employees are more cynical about the meaningfulness of their job [51] and such reduce task importance, or significance, depends on how many other overqualified peers work in the same context [52]. We believe that low levels of importance to one’s job may be, for young overqualified employees, a way to reduce the cognitive dissonance between their qualification and skill’s usage and this may help in maintaining higher level of mental health. Therefore, future research should study the boundary conditions of the relationship between the antecedents here described (role conflict, role overload, and overqualification) and the misaligned wellbeing patterns.

Future studies might also investigate other potentially relevant antecedents, or moderators, of the mis/alignment between job satisfaction and mental health. In particular, following Danna and Griffin [4] and Nielsen et al. [53], it might be interesting to examine the sector of employment, in particular if it involves hazardous and stressful work settings (requiring for instance, night shifts or traveling), job resources as job autonomy, and also HR practices and social support. Literature has in fact showed that such factors may increase or decrease job satisfaction and wellbeing [4,53], but it should be examined if they differentially affect job satisfaction and mental health, also in relation to the family status of the employees (single, married, with children) and family history of mental health.

Finally, another major point concerns the sample being studied. The sample is composed of so-called millennials, thus, a very specific subgroup. These employees might differ in their general health (both mental and physical), wellbeing, satisfaction, etc., from other employees. As a group, millennials are in between twenties and late thirties, thus they do have a better physical health than older generations. Nevertheless, their lower tolerance to frustrations and their need to deal and face the economic crisis (initiated on 2008), which results in fewer career opportunities, may have as an effect a poorer level of mental health, especially in minor symptoms such as anxiety, life dissatisfaction, etc. Another specific situation of the millennials is that they face the transition from school to work in a situation that is in many cases not favorable. The support from their families, the resignation to have precarious/flexible jobs, to get incomes for subsistence or searching for jobs abroad, are some of the different ways of coping with the complex and difficult situation that millennials are facing during the actual economic crisis period.

## 5. Limitations and Practical Implications

One of the potential limitations of the current study is related to the sample, which is limited to young Spanish employees. To make better generalizations about the four wellbeing patterns, it is necessary to document their occurrence in other contexts. However, the sample was representative of all the regions of Spain, and the independent variables (role stress, job importance, and overqualification) that we tested in this research might be applicable to millennials in other contexts, which could make the generalization of these research findings more robust. Therefore, this limitation may be at least partially neutralized because the procedures and variables we used are applicable to millennials in other contexts. In addition, to test the external validity of the study, it would be useful to replicate it with millennials in other countries.

Another limitation is related to the measurement of job satisfaction and mental health. In job satisfaction measurement, cognitive/subjective biases may affect employees’ evaluations of their satisfaction. Similarly, we assessed mental health by using the General Health Questionnaires (GHQ) in terms of a specific time period: ‘*during the past few weeks*…’. However, events occurring “weeks ago” may be poorly recalled, and, therefore, induce some possible inaccuracy in mental processing [3]. However, the measurements of both job satisfaction and mental health are based on well validated and accepted instruments, and so we expect cognitive/personal biases and inaccuracy in mental processing to have little or no impact on the validity of the current research findings.

One of the main aims of organizational psychology is to improve employees’ wellbeing. In this regard, our taxonomical approach provides relevant empirical evidence, facilitating the achievement of this endeavor. First, by combining job satisfaction and mental health, this study maps synergistic but also misaligned wellbeing patterns. Second, our study also provides valuable information of some personal and organizational variables related to them. In this way, our study informs organizational psychologists of when they may be improving at the same time job satisfaction and mental health, but also when this might not happen, creating misaligned wellbeing patterns instead. Thus, an important implication of our study is the provision of a useful wellbeing-pattern taxonomy from where to study and improve employees’ wellbeing.

We also identify implications for other stakeholders. For instance, our results show that it would be worthwhile for organizations to find mechanisms to track and ensure the fulfillment of their commitments to millennials. Our results show, in fact, that only a small portion of employees are in the optimal job satisfaction and mental health category, whereas larger portions are in the unsatisfied-unhealthy and misaligned patterns. At the same time, organizations should carefully consider HR policies, such as staffing, to establish mechanisms to avoid phenomena such as role ambiguity, role conflict, role overload, and overqualification. These organizational and personal phenomena have been shown to have toxic effects on both job satisfaction and mental health.

Third, the results show that job importance is an important mechanism for a sustainable young workforce. In our study, young employees who reported having high job importance were characterized by being satisfied and healthy. Therefore, we argue that managers and employers should increase job importance by providing incentives related to job satisfaction and mental health. Often the jobs available for youngsters are “poor” overqualified and in some cases precarious. Thus, it is important that the companies enhance the meaning of work for youngsters offering jobs that are valuable and meaningful. This is the type of “incentives” that may make work more meaningful for youth and less toxic, dissatisfying and unhealthy.

## 6. Conclusions

At the beginning of this paper, we noted that the relationship between job satisfaction and mental health is mainly considered harmonious, and that there is scarce research about the anomalous/misaligned patterns between these two variables. The main aim of this study was to extend the relationship between job satisfaction and mental health by identifying four patterns: satisfied-healthy, satisfied-unhealthy, unsatisfied-healthy, and unsatisfied-unhealthy. This taxonomy seems to provide a valid, interesting, and useful way to study employees’ wellbeing by considering their job satisfaction and mental health. It is our hope that addressing this more extended pattern of relationships between the two variables will lead to a possible resolution of the satisfied-healthy conundrum. Thus, the unsatisfied-healthy or satisfied-unhealthy profiles should be the targets of future research. Moreover, this research has contributed to identifying some organizational and personal antecedents that influence and differentiate the four patterns. Future research will need to study how stable or dynamic these patterns are over time, and what their consequences are in the long run. This knowledge will help us to create more effective interventions so that organizations can assist millennials in moving toward a more positive and optimal job satisfaction level and assess its contribution to health and vice-versa.

## Figures and Tables

**Table 1:** Bivariate correlations and descriptive statistics of the job satisfaction-mental health pattern predictors.

Variables	*M*	*SD*	1	2	3	4	5	6	7	8	9	10	11
1. Age	25.21	3.40	-	−0.02	0.21 **	−0.00	0.02	0.09 *	0.08 *	0.02	0.07	0.12 **	−0.03
2. Gender	0.48	0.50		-	0.11 **	0.06	−0.09 *	0.04	0.08 *	0.07 *	−0.08 *	0.02	0.00
3. Type of contract	0.58	0.50			-	−0.01	−0.05	0.02	0.01	−0.09 *	0.05	0.14 **	0.05
4. Type of sector	0.18	0.38				-	−0.13 **	0.03	0.01	−0.00	0.07 *	0.09 *	−0.04
5. Overqualification	0.22	0.41					-	−0.02	0.09 *	0.04	−0.03	−0.20 **	-0.04
6. Role overload	2.91	0.97						(0.82)	0.37 **	0.07	0.06	0.05	−0.15 **
7. Role conflict	2.78	0.99							(0.75)	0.25 **	−0.06	−0.12 **	−0.29 **
8. Role ambiguity	2.05	0.80								(0.80)	−0.28 **	−0.46 **	−0.25 **
9. Job importance	4.14	0.51									(0.89)	0.50 **	0.16 **
10. Job satisfaction	3.68	0.73										(0.94)	0.26 **
11. Mental health	3.67	0.83											(0.76)

Internal alpha estimates are in parenthesis * *p* = 0.05; ** *p* < 0.01. (two-tailed).

**Table 2:** Discriminant analysis of the four patterns of relations between job satisfaction and mental health with job importance, role ambiguity, role conflict, role overload, and overqualification as discriminant variables.

Variables/Discriminant Function Statistics	Means (Standard Deviations) of Wellbeing Patterns				Standardized Discriminant Function Coefficients ^a^		
	Cluster 1	Cluster 2	Cluster 3	Cluster 4			
	Unsatisfied-Unhealthy (*n* = 258)	Unsatisfied-Healthy (*n* = 208)	Satisfied-Unhealthy (*n* = 194)	Satisfied-Healthy (*n* = 112)	Function 1	Function 2	Function 3
Covariates							
Gender ^b^^,^^c^	0.48 (0.50)	0.44 (0.50)	0.52 (0.50)	0.45 (0.50)	0.10	0.07	−0.03
Sector ^c,d^	0.21 (0.41)	0.13 (0.33)	0.17 (0.38)	0.22 (0.42)	−0.07	0.06	−0.04
Type of contract ^c,e^	0.54 (0.50)	0.58 (0.50)	0.61 (0.49)	0.64 (0.48)	−0.06	0.08	0.15
Age	24.96 (3.43)	24.99 (3.33)	25.85 (3.31)	24.81 (3.56)	−0.07 (−0.09)	0.21 (0.12)	0.77 (**0.77**)
Discriminant variables							
Job importance	3.96 (0.48)	3.97 (0.50)	4.29 (0.44)	4.52 (0.33)	−0.79 (**−0.73**)	0.25 (0.24)	−0.07 (−0.17)
Role ambiguity	2.33 (0.77)	2.12 (0.78)	1.86 (0.66)	1.51 (0.64)	0.65 (**0.50**)	0.17 (0.03)	−0.12 (−0.13)
Role conflict	3.09 (0.88)	2.53 (0.97)	2.82 (0.93)	2.39 (1.12)	0.29 (0.13)	0.84 (**0.78**)	−0.16 (**−0.43**)
Role overload	3.03 (0.88)	2.77 (0.97)	3.03 (0.93)	2.59 (1.07)	0.14 (0.14)	0.54 (0.23)	0.48 (**0.59**)
Overqualification	0.23 (0.42)	0.30 (0.46)	0.17 (0.38)	0.13 (0.34)	0.19 (0.22)	−0.32 (**−0.41**)	0.15 (0.18)
Significance of function					0.01	0.01	0.05
Canonical correlation					0.50	0.26	0.13
Eigenvalue					0.32	0.07	0.02
Explained variance					79.1%	17.1%	3.9%
Centroid of:							
Cluster 1					0.52	0.20	−0.09
Cluster 2					0.27	−0.39	0.06
Cluster 3					−0.42	−0.22	0.16
Cluster 4					−1.15	−0.13	−0.20

Note: *n* = 772 after listwise deletion of cases with missing data. ^a^ In parentheses are the coefficients of a stepwise solution that included only variables entered at the 0.05 significance level (coefficients higher than 0.30 are in boldface). The stepwise criterion was minimization of the overall Wilks’ lambda. ^b^ Gender: Coded 0 = male, 1 = female. ^c^ The group mean of the dichotomous/dummy variables indicates the proportion of the higher coded category. ^d^ Sector: Coded 0 = private, 1 = public. ^e^ Type of Contract: Coded 0 = temporary, 1 = permanent.

## Appendix A

**Table A1:** Constructs Definitions and their relationship with employees’ wellbeing.

Constructs	Definitions	Relationships with Employees’ Wellbeing
Job satisfaction	Job satisfaction is an individual’s attitude toward the job, that is, an overall evaluative judgment about one’s job that is caused by affective experiences on the job and (cognitive) beliefs about the job [54].	A recent review of quantitative studies show that job satisfaction is the most common conceptualization of employee wellbeing [55]
Mental health	We operationalize the definition of the health sub-dimension of wellbeing proposed by Danna and Griffin [4] in terms of general positive mental health self-reported by employees [36].	Danna and Griffin [4] consider mental health to be a sub-component of wellbeing at work
Role conflict	We define role conflict as parties’ contradictory expectations about aspects of a single role or between different roles [15,56].	Studies indicate that when employees are exposed to conflicting and ambiguous roles, they experience job dissatisfaction and low mental health [18,19].
Role ambiguity	We define *role ambiguity* as lack of sufficient information or uncertainty about expectations and actions to fulfill a role/job [15,56]	
Role overload	We define *role overload* as lack of the personal resources that an individual needs to fulfill multiple roles, commitments, obligations, or requirements demanded by the work [15]	Research findings on the impact of role overload on employee job satisfaction and mental health are inconsistent. Some scholars, for instance, show that role overload correlates with low job satisfaction [57] and low mental health (such as experiencing fatigue and tension) [38]. However, in other research, Cooke and Rousseau [38] indicate that role overload does not affect the job satisfaction of employees engaged in multiple roles.
Job importance	Job importance refers to the level of personal significance and value an employee associates with various facets of the job (extrinsic, intrinsic and social) [23,58].	We argue that jobs that provide employees with intrinsic, extrinsic, and social job importance facets enhance job satisfaction and mental health.
Overqualification	We define overqualification as employees’ perceptions of having excess education, knowledge, abilities, and skills, compared to the requirements of the job [59]	Scholars indicate that overqualified employees enjoy greater satisfaction with life [28], which correlates with better mental health [10].
